# Closing-wedge high tibial osteotomy: survival and risk factor analysis at long-term follow up

**DOI:** 10.1186/1471-2474-12-46

**Published:** 2011-02-14

**Authors:** Turgay Efe, Gafar Ahmed, Thomas J Heyse, Ulrich Boudriot, Nina Timmesfeld, Susanne Fuchs-Winkelmann, Bernd Ishaque, Stefan Lakemeier, Markus D Schofer

**Affiliations:** 1 Department of Orthopaedics and Rheumatology, University Hospital Marburg, Baldingerstrasse, 35043 Marburg, Germany; 2 Department of Orthopaedics, Sankt Elisabeth Hospital, Stadtring Kattenstroth 130, 33332 Gütersloh, Germany; 3 Institute of Medical Biometry and Epidemiology, Philipps-University Marburg, Bunsenstrasse 3, 35037 Marburg, Germany; 4 Clinic and Polyclinic for Orthopaedic Surgery, University Hospital Giessen, Paul Meimberg Str. 3, 35392 Giessen, Germany

## Abstract

**Background:**

Closing-wedge high tibial osteotomy (HTO) is successful for the treatment of medial osteoarthritis with varus malalignment. Preoperative risk factors for HTO failure are still controversial. The aim of this study was to elucidate the outcome and assess the influence of risk factors on long term HTO survival.

**Methods:**

199 patients were retrospectively studied with a mean follow-up period of 9.6 years after HTO. HTO failure was defined as the need for conversion to TKA. Survival was analyzed with the Kaplan-Meier method. Knee function was evaluated by the Hospital for Special Surgery (HSS) score. HTO-associated complications were also assessed. Univariate, multivariate, and logistic regression analysis were performed to evaluate the influence of age, gender, BMI, preoperative Kellgren-Lawrence osteoarthritis grade, and varus angle on HTO failure.

**Results:**

39 complications were recorded. Thus far, 36 HTOs were converted to TKA. The survival of HTO was 84% after 9.6 years. Knee function was considered excellent or good in 64% of patients. A significant preoperative risk factor for HTO failure was osteoarthritis, Kellgren-Lawrence grade >2.

**Conclusion:**

HTO provides good clinical results in long-term follow-up. Preoperative osteoarthritis Kellgren-Lawrence grade >2 is a significant predictive risk factor for HTO failure. Results of HTO may be improved by careful patient selection. Complications associated with HTO should not be underestimated.

## Background

Closing wedge high tibial osteotomy (HTO) is an accepted procedure in the treatment of medial knee osteoarthritis with varus malalignment. Since Jackson and Waugh [1] initially described osteotomy below the tibial tubercle, various modifications of HTO have been published [2-7]. The fundamental goal of HTO is to partially offload the medial compartment and to realign the knee into valgus. Several studies have shown satisfactory short and medium term success; however, valgus-producing tibial osteotomy gradually deteriorates with time [8-10].

Ideal candidates for HTO are young and active patients who are not suitable for total knee arthroplasty (TKA) [11]. Even though TKA is a popular and well-established method, younger age was associated with an increased risk of revision [12]. Despite the fact that HTO results show its effectiveness, great debate on risk factors influencing HTO survival is still on-going. Various factors including age, gender, BMI, activity level, and varus angle are claimed to affect the duration of survival [13-18]. Selecting the ideal patient and identifying risk factors that may affect osteotomy longevity is important to obtain satisfactory results with HTO. The aim of this study was to determine HTO failure requiring conversion to TKA, including risk factors at long-term follow up. Furthermore, potential complications and clinical outcome of high tibial osteotomy were evaluated.

## Methods

239 cases of closing-wedge osteotomy were performed between 1984 and 2001 at our institution. 13 (5.5%) patients were lost to follow-up. 27 (11%) patients were too ill or refused to participate in clinical and radiological assessment. With these patients, telephone interviews were performed to obtain information on further operations following HTO. 199 (83%) patients were therefore clinically and radiologically assessed. This study was approved by the ethics committee of the University Hospital Marburg and informed consent was obtained from all patients. This project was performed in accordance with the Helsinki Declaration, and with local legislation.

Patient demographics and clinical characteristics are illustrated in Table 1. The left knee was involved in 104 (52.2%) cases and the right in the remaining 95 (47.8%) cases. The average age at HTO was 54 ± 8 (25-72) years. The mean time of follow-up was 9.6 (1-18) years including 110 (55.3%) men and 89 (44.7%) women. A previous partial arthroscopic meniscectomy (less than one third) was performed in 51 (25.6%) cases.

**Table 1:** Patient demographics and baseline characteristics at the time of closing wedge osteotomy.

Age, mean (SD)	54 (8)
Men	110
Women	89
BMI, mean (SD)	28 (4)
20-24.9	36
25-29.9	96
30-34.9	52
35-39.9	10
>40	5
Varus angle (°), mean (SD)	6° (3)
Kellgren-Lawrence grading	
Grade 1	98
2	96
3	5
Smoker	21

The indication for HTO was symptomatic medial compartment osteoarthritis with varus malalignment. Preoperative radiographs included the full leg and standard short anteroposterior views in the standing position with the knee in full extension, as well as lateral and merchant views. The severity of osteoarthritis was scored according to the Kellgren-Lawrence system (0 normal, 4 severe [19]). Varus angle was measured using mechanical axes of the femur and the tibia (hip-knee-ankle angle) obtained on full length, full weight-bearing standing radiographs.

All patients were underwent a lateral closing-wedge technique [4]. Fibular transection was performed at the junction of the middle and distal thirds [20] through a separate incision. A transverse incision with the patient in supine position was performed for the tibial osteotomy. The peroneal nerve was exposed and protected. The osteotomy was performed below the tibial tuberosity, leaving the medial cortex intact. Bone wedge size was based on the preoperative calculations from the long leg standing radiograph. A laterally-based wedge of bone was removed and the osteotomy was fixed with an AO-plate. Postoperative early active movement and physiotherapy were allowed. No casts were applied. Partial weight bearing using 2 crutches for 6 weeks was allowed until bony union was reached. Control standing X-rays were taken 6 weeks, 6 months, and 1 year after surgery as well as at time of latest follow-up.

The correlation between the conversion of HTO to TKA and possible risk factors including age, gender, body mass index (BMI), Kellgren-Lawrence grade of osteoarthritis, and varus angle were determined. Smoking was defined as a daily consumption of three or more cigarettes [21]. All patients underwent physical examination and the application of a clinical score by co-author GA, an orthopaedic resident. Knee function was evaluated by the Hospital for Special Surgery (HSS) score [22], consisting of a questionnaire and physical examination. The items are clustered into six categories which include pain, function and range of motion, muscle strength, flexion deformity, and instability. A maximum score is 100 points. 85-100 points are excellent, 70-81 good, 60-69 fair, and <60 poor. Knee ROM was measured using a goniometer [23]. An independent specialist in radiology evaluated the diagnostic images. All of the osteotomies were performed by two experienced knee surgeons. The operating surgeons were not involved in the clinical assessment.

HTO failure was defined as the need for conversion to TKA. Survival analysis was performed applying the Kaplan-Meier method. Potential risk factors (age, gender, BMI, Kellgren-Lawrence grade of osteoarthritis, and varus angle) were calculated by the univariate Log-Rank test. Cox regression models were used in both univariate (single risk factors) and multivariate (combinations of risk factors) analysis. Smoking as a risk factor for complications was calculated by Fisher's exact test. A p-value of < 0.05 was considered to be statistically significant.

## Results

**Figure 1:**
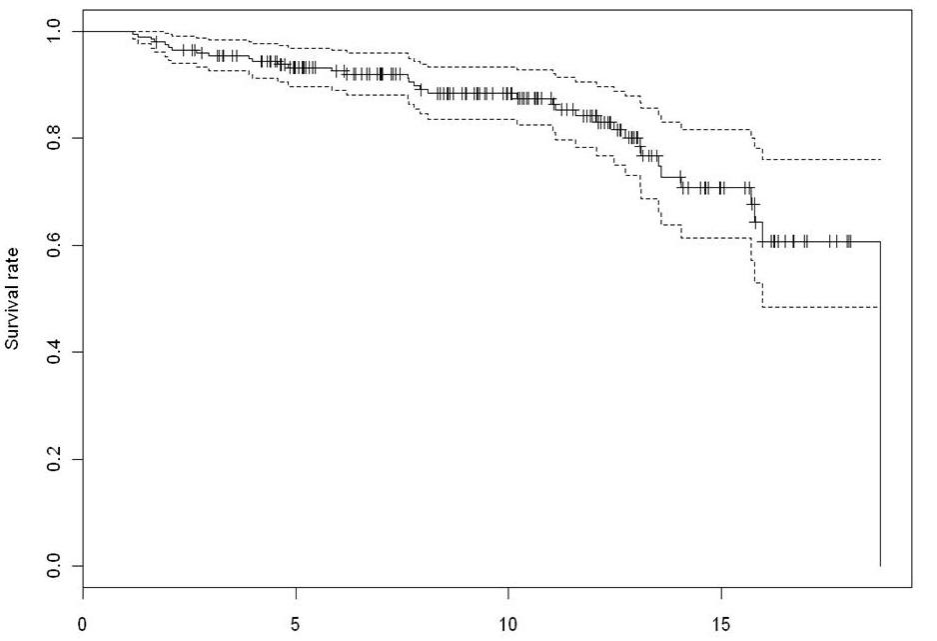
**Survival rate analysis after HTO using the Kaplan-Meier method**. The conversion to TKA after HTO was set as the endpoint. 93% of patients at 5 years, 84% of patients at 9.6 years and 68% at 15 years after HTO did not require conversion to TKA.

At latest follow-up, 36 (16%) HTOs had been converted to TKA. The 5-year, 9.6 year, and 15-year HTO survival rates as determined by Kaplan-Meier analysis were 93%, 84%, and 68% (Figure 1). 54 patients had excellent results, 74 good, 51 fair and 20 poor. Pain was absent in 74 patients, mild in 66 cases, and severe in 58 cases (in motion but not at rest). No patient complained of continuous pain.

Partial arthroscopical meniscectomy prior to HTO did not influence HTO survival as shown by a multivariate Cox model (p = 0.65) and the log-rank test (p = 0.71).

Using the univariate and multivariate Cox regression model, age had no influence on HTO survival (p = 0.32 and p = 0.31, respectively). There was no significant difference between the survival rate of men and women using the multivariate Cox model (p = 0.32) and the Log-Rank test (p = 0.31). Univariate and multivariate Cox regression models found the preoperative varus angle not to be a significant factor (p = 0.8 and p = 0.84, respectively). The correlation between BMI and HTO failure was not significant using the univariate (p = 0.2) and multivariate (p = 0.11) Cox regression model as well as Log-Rank test (p = 0.45). Statistical significant correlation between the preoperative Kellgren-Lawrence osteoarthritis grade >2 and HTO failure could be detected with the use of both univariate (p = 0.003) and multivariate (p = 0.01) Cox regression models.

38 (19%) complications were recorded: 8 cases of deep vein thrombosis requiring low molecular weight heparin and compressive therapy; 1 vascular injury requiring surgery; 6 cases of peroneal palsy left with permanent deficit; and 3 superficial wound infections which responded to antibiotics. 9 patients had non-union of the osteotomized tibia and 8 of the fibula which required reostheosyntheses with bone grafting. Comparing obese and non-obese patients, no significant difference in complications was noted (p = 0.37). 6 of the 21 smokers had postoperative complications (3 non-union of the tibia, 2 non-union of the fibula, and 1 deep vein thrombosis). There was no significant difference in complications between smokers and non-smokers (p = 0.54).

## Discussion

We aimed to evaluate the clinical outcome of HTO and to assess potential risk factors that may influence its longevity. There were several limitations to our study, including its retrospective design and lack of control group. There were a significant number of patients who were lost to follow-up, although over 80% of patients could be included, which is satisfactory when compared with other studies in this area [18,24].

Although there have been several studies on closing-wedge HTO, there is great variability in the results. Differences in patient outcome may be caused by wide heterogeneity among studies (e.g. different techniques and evaluation systems, varying degrees of deformity, and varus angle). Pooling the results is a challenge, as described by Amendola et al. [25] in a systematic review. There are few randomized controlled trials; well designed studies should include larger numbers to generate a higher quality of evidence. Long-term results are also needed for more solid conclusions. Thus, we conducted this study in order to select the ideal patient for HTO and improve the understanding of potential associated complications.

With the increasing numbers and success of TKA [26], fewer HTOs have been performed in the last several decades [27]. Although Brouwer et al. failed to show in a systemic review that HTO was more effective than conservative treatment [28], it is an accepted method for medial gonarthritis with varus malalignment [29].

The complication rate of HTO is between 5.6% and 34% [30-32], including non-union and peroneal palsy. In our study, the 19% complication rate is within the range of published results [33]. We performed all osteotomies below the tibial tuberosity, with 9 cases of non-union. Non-union of the tibia is an uncommon complication after closing-wedge HTO, but is more frequently found in osteotomies below rather above the tibial tubercle (14% vs 3.6%) [33]. The risk of peroneal palsy is the most reported neurovascular complication after HTO [34]. Wootton et al. [35] showed that the majority of peroneal damage occurred when fibular osteotomy is performed 8-15 cm below the fibular head - a zone that should be avoided when performing fibular osteotomy. Even when fibular transection was performed at the junction of the middle and distal thirds, 6 cases of peroneal palsy were noted. Peroneal palsy was a substantial contributing factor to patient dissatisfaction in our cohort.

Survival rate analysis revealed an 84% survival at 9.6 years follow-up, which is confirmed in other studies (75%-98%) [36-38]. Clinical outcome was also considered excellent or good in 64% cases 9.6 years postoperatively, similar to previous studies [31,36,39]. Results appear to gradually deteriorate after ten years [10,13,25].

Patient age is a crucial criterion for HTO indication. In our study, age was not a significant predictive factor for HTO failure. Contrary to our findings, Gstöttner et al. [8] and Trieb [18] demonstrated that age significantly influenced HTO survival.

As observed by other authors [11,14,37,40], a significant association exists between preoperative Kellgren-Lawrence osteoarthritis grading and HTO failure. Patients with a higher grade of osteoarthritis (>2) may be better treated with arthroplasty. Huang et al. [41] reported that a large preoperative varus angle is influences HTO survival. We did not find this to be true in our cohort. A possible explanation for this discrepancy may be that in our patients, there was only a moderate average preoperative varus angle (6 degrees). However, two studies with similar preoperative varus angles (6° [37] and 6.5° [14]) confirmed our findings. Obesity is a growing problem in industrialized countries; Some studies have reported that HTO in obese patients is associated with early failure [15,31,42]. In our cohort, significant differences between obese and non-obese patients was not found [43]. In a systematic review and meta-analysis, Blogojevic et al. [44] demonstrated female gender to be both a risk factor for osteoarthritis, and associated with severe OA. In our data, gender was not associated with HTO failure.

For several years, the lateral closing wedge HTO was the preferred treatment approach for patients with medial knee osteoarthritis. Due to some disadvantages of this technique, the medial opening wedge HTO regained popularity, since improvements in operative approach and special implants were developed [45,46]. There is only one prospective, randomized study comparing lateral closing wedge and medial opening wedge osteotomies [47]. At one year follow-up, both techniques reduced pain and improved knee function, but the difference was not significant.

A further option for treatment of unicompartimental gonarthrosis is unicompartimental arthroplasty (UKA). Even if there are differences in the indications for the two procedures, some patients can be treated either by HTO or UKA. Only a few studies compare the clinical outcome of UKA versus closing wedge HTO [48-50], and only two were randomized controlled studies [51,52]. Data comparing UKA with HTO appear to support UKA over HTO. However, Brouwer et al. [28] stated in their meta-analysis that there is silver evidence for no significant difference in pain, knee function, and gait after HTO compared to UKA, and that HTO causes more complications than UKA.

## Conclusion

HTO has a favorable outcome at 9.6 years of follow-up. A significant risk factor for HTO failure was preoperative Kellgren-Lawrence osteoarthritis grade >2. In order to improve the durability of HTO, appropriate patient selection is necessary. Surgeons should be aware that HTO is not without complications.

## Competing interests

The authors declare that they have no competing interests.

## Authors' contributions

TE drafted the manuscript and participated in its design and coordination; GA investigated, followed, and clinically managed patients; NT performed the statistical analysis; TJH, SFW, SL and BI participated in analysis and interpretation of data; MDS and UB initiated the study and participated in its design and coordination. All authors read and approved the final manuscript.

## Pre-publication history

The pre-publication history for this paper can be accessed here:

http://www.biomedcentral.com/1471-2474/12/46/prepub
